# A Wireless LC Sensor Coated with Ba_0.9_Bi_0.066_TiO_3_ for Measuring Temperature

**DOI:** 10.3390/s150511454

**Published:** 2015-05-18

**Authors:** Milan Radovanovic, Bojana Mojic-Lante, Katarina N. Cvejin, Vladimir V. Srdic, Goran M. Stojanovic

**Affiliations:** 1 Faculty of Technical Sciences, University of Novi Sad, Trg Dositeja Obradovica 6, Novi Sad 21000, Serbia; E-Mail: sgoran@uns.ac.rs; 2 Saint-Gobain R&D Center, 9 Goddard road, Northborough, MA 01532, USA; E-Mail: bojana.lante@saint-gobain.com; 3 Faculty of Technology, University of Novi Sad, Bulevar Cara Lazara 1, Novi Sad 21000, Serbia; E-Mail: cvejin.katarina@gmail.com; 4 Instytut Technologii Elektronowej, Ul. Zablocie 39, Krakow 30-701, Poland; E-Mail: srdicvv@uns.ac.rs

**Keywords:** LC sensor, wireless, BBT ceramic, measuring temperature

## Abstract

This paper presents a passive LC wireless sensor for measuring temperature. The sensor is designed as a parallel connection of a spiral inductor and an interdigitated capacitor and it was fabricated in a conductive layer using LTCC (Low Temperature Co-fired Ceramic) technology. The inderdigitated capacitor electrodes were coated with a thin film of bismuth doped barium titanate (Ba_0.9_Bi_0.066_TiO_3_), whose permittivity changes with temperature, which directly induces changes in the capacitance of the interdigitated capacitor and consequently changes the resonant frequency of the sensor. The measurements of *S*-parameter of the sensor were performed using a Vector Network Analyzer (E5071B, Agilent Technologies, Santa Clara, CA, USA), whose port was connected to the antenna coil that was placed around the sensor in order to be able to wirelessly detect temperature, in the temperature range from 25 °C to 165 °C.

## 1. Introduction

Temperature measurements are very significant for successful functioning of numerous electronic devices which surround us in everyday life. Furthermore, almost every industrial process requires temperature measurements in the range of room temperature up to a several hundred degrees Celsius. Various methods for measuring temperature can be used, naming: thermocouples, resistive temperature devices (RTDs and thermistors), capacitive sensors, infrared radiators, bimetallic devices, liquid expansion devices, and change-of-state devices [1,2,3].

Many papers related to the applications of resonant inductive-capacitive (LC) circuit based sensors have been published in the last decade, because these sensors eliminate the need for onboard power and physical connections [4,5]. Due to a small size and stable characterization of the LC sensors, they are particularly suitable for transmitting power to short distances in industrial harsh environments. So far, a significant amount of research has managed to advance the LC based passive wireless sensing technologies and extend their applications in many areas, including pressure sensor [6], humidity sensors [7,8], and temperature sensors [9]. 

The trends of miniaturization, increased reliability and high ambient operating temperatures for electronic circuits have driven the deployment of ceramic substrates and packages. Among other technological approaches, LTCC (Low Temperature Co-fired Ceramic) have proven their superior performance in a variety of applications. These comprise high temperature automotive, highly reliable medical applications and RF modules for wireless communication [10]. Capacitors prepared from barium titanate based ceramics are used often in those systems due to their excellent dielectric properties. In addition, many commercial multilayer capacitors contain a certain amount of bismuth because of its significant effect on lowering the sintering temperatures. Bismuth is also proved to be able to enhance the magnitude of positive coefficients of resistance and dielectric properties. Therefore, adding bismuth seems promising since the dielectric properties of barium titanate based ceramics are very sensitive to both microstructure and defect chemistry of the materials, which are strongly influenced by processing parameters such as chemical composition and sintering conditions [11]. 

This paper presents a passive LC sensor for temperature measurements. The sensor was fabricated in LTCC technology, and barium bismuth titanate (BBT) thin film was coated over electrodes of an interdigitated capacitor. Hence, the temperature changes the permittivity of a BBT oxide layer which directly induces changes in the capacitance of the interdigitated capacitor. *S*-parameters of the sensor were measured using an instrument Vector Network Analyzer E5071B (Agilent Technologies), with an antenna coil placed around the sensor and connected to the port of this instrument. The sensor presented in this paper can operate in a temperature range from 25 °C to 165 °C. The advantage of our sensor is that it is passive, and there is no problem with the battery lifetime. Moreover, the proposed sensor design does not use vias and thus saves material, reduces fabrication time and most importantly, reducse the ultimate cost of the sensor.

## 2. Coating of Interdigitated Capacitor Electrodes with BBT Film

### 2.1. Sensor Design

**Figure 1:**
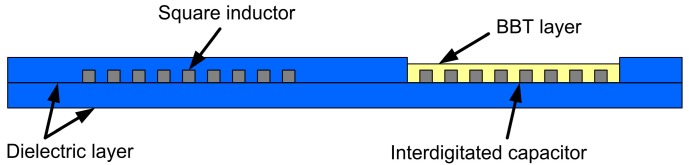
The inductive-capacitive (LC) sensor cross section.

**Figure 2:**
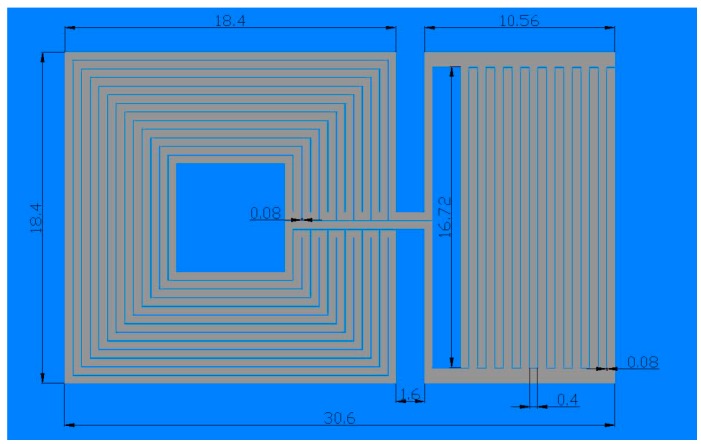
The dimensions of the LC sensor.

The cross section of the sensor presented in this paper is a planar resonant circuit, made of a square inductor, and an interdigitated capacitor (Figure 1). This LC sensor was realized that the inductance of the sensor remains constant. The inductive part was covered with dielectric layer. Contrary to this, the capacitance of the interdigitated electrode system was changed with the variation of the permittivity of the medium (exposed through the small window from the top side of the sensor). This would cause the shift of sensor’s resonant frequency. Sensor dimensions, shown in Figure 2, were expressed in millimeters. The interdigitated capacitor had 18 sets of electrodes (fingers) of 16.72 mm in length. The conductive lines width for the inductor and the capacitor was 0.4 mm. The spacing between two adjacent electrodes or capacitor’s fingers was 0.08 mm.

The interdigitated capacitor electrodes of the sensor were coated with nanocrystalline bismuth doped barium titanate—Ba_0.9_Bi_0.066_TiO_3_ (BBT) film of a thickness of around 10 mm. BBT was chosen as a material with close to linear permittivity change with temperature in a desired temperature range [12], while in the same time being environmentally friendly (not containing Pb).

### 2.2. Powder Preparation and Characterization

**Figure 3:**
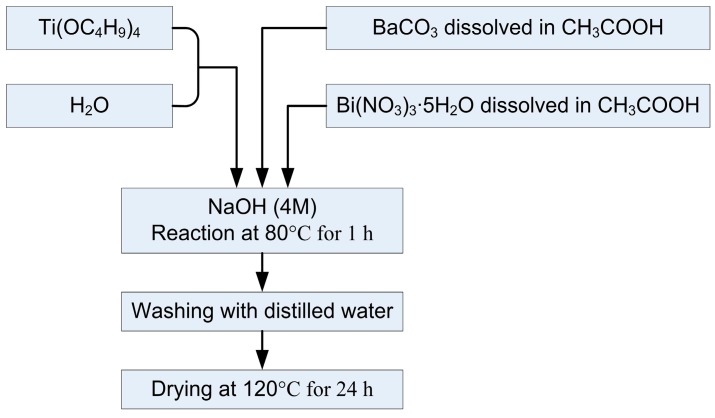
Scheme of the bismuth doped barium titanate (BBT) powder synthesis procedure.

Nanocrystalline bismuth doped barium titanate (Ba_0.9_Bi_0.066_TiO_3_, BBT) powder was synthesized as described in more details further in the paper [13]. Powder preparation process is shown schematically in Figure 3. Controlled hydrolysis of titaniumbutoxide (Ti(OC_4_H_9_)_4_, Fluka, Buchs, Switzerland) with distilled water, and then further reaction of the formed amorphous titanium hydroxide gel particles with Ba^2+^ and Bi^3+^ ions under strong alkaline solution reaction conditions (pH > 13) was performed. Barium carbonate (BaCO_3_, Merck, Darmstadt, Germany) and bismuth (III) nitrate pentahydrate (Bi(NO_3_)_3_∙5H_2_O, Sigma Aldrich, St. Louis, MO, USA) dissolved in acetic acid were used as precursors for corresponding ions. The reaction between the formed titanium hydroxide particles and corresponding ions was carried at 80 °C for 1 h. Powders were washed in distilled water and dried at 120 °C for 24 h. Obtained powder was used as a functional component in the preparation paste. 

In order to confirm the phase composition of the synthesized powder, X-ray diffractometer Bruker D8 (Bruker AXS GmbH, Karlsruhe, Germany), advanced with Ni-filtered Cu Kα radiation, λ = 0.15406 nm, equipped with Sol-X detector, was used. The measurement was conducted with the step size of 0.03° and sampling time of 3 s per step. 

### 2.3. Preparation of the BBT Paste and Thin Film

**Figure 4:**
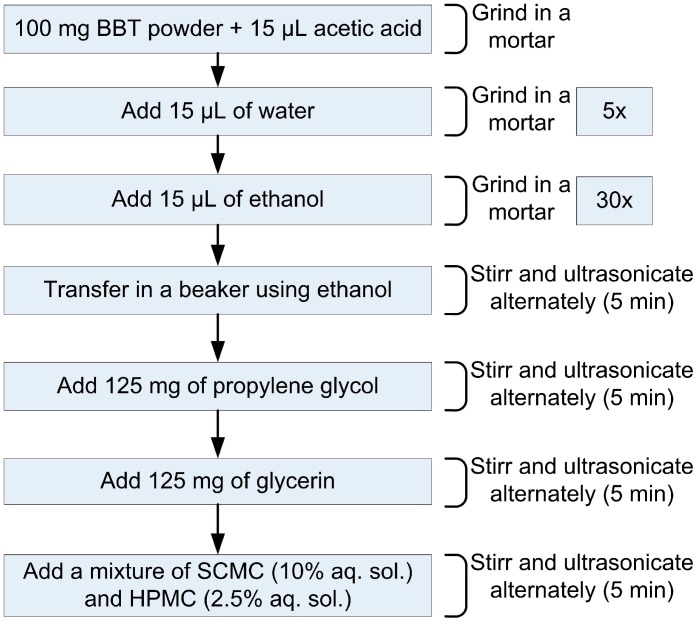
Scheme of the paste preparation from nanocrystalline BBT powder.

The main aim during the fabrication of the thin film was to prepare an environmentally-friendly paste. Accordingly, the components used for the preparation of the paste were the following: (I) BBT powder as a functional component; (II) propylene glycol (Centrohem, Stara Pazova, Serbia), absolute ethanol (Merck, Germany) and distilled water as solvents; (III) cellulose derivates—sodium carboxymethylcellulose (SCMC, Sigma Aldrich) and hydroxypropylmethylcellulose (HPMC, K100M Colorcom LTD, Dartford Kent, UK) for optimization of the paste rheology; (IV) glycerine (88%–86%, Zorka, Šabac, Serbia) as a plasticizer and (V) glacial acetic acid (Centrohem, Stara Pazova, Serbia) as a surfactant. The paste was fabricated similarly to the procedure intended for screen printing pastes for dye-sensitized solar cells [14]. The preparation procedure is schematically presented in the Figure 4. Namely, 100 mg of BBT powder was grinded in the agate mortar, with the addition of acetic acid, water and ethanol in small amounts (15 μL), and the total volume of 15 μL of acetic acid, 75 μL of water, and 450 μL of ethanol was added. Grinded powder was transferred in a glass vessel using excess of ethanol, and alternately stirred and ultrasonicated for 5 min. Afterwards, the rest of the components were added (in the following order: 125 mg of propylene glycol, 125 mg of glycerine, the mixture of 10% solution of SCMC in water and 2.5% HPMC solution in water—in total 2 g) and solution was stirred and ultrasonicated upon addition of each. During the preparation, the amount of glycerine was found to be important, since the films with the smaller amount of glycerine were not fixed to the substrate, but peeled off in one peace. The particular mixture of SCMC and HPMC was selected based on the study of Sanson *et al.* [15], where among a few cellulose derivates examined, the mixture of those two gave the best results in terms of elasticity and thus the microstructure and crack-free surface of the obtained film. The prepared paste was manually deposited on the capacitive part of the LC sensor prepared by LTCC technology and after drying at 80 °C for 30 min, the sensor was thermally treated at 550 °C for 1 h in air. Upon dielectric measurements, the sensor was additionally thermally treated at 800 °C for 1 h, in order to examine potential impact of sintering temperature on the performance of the fabricated sensor. Dielectric behavior of bismuth doped barium titanate (BBT) with various Bi^3+^/Ba^2+^ ratios was investigated in [13], in the temperature range 25–190 °C. Results obtained in [13] implied that barium bismuth titanate could be used as a sensitive layer of a passive LC temperature sensor. In the present study it is shown that BBT layer screen printed over an interdigitated capacitor can be used as a temperature sensitive layer in passive LC temperature sensors.

The structural characteristics of the film after thermal treatment were examined using the optical microscope MOTICAM 2300 (Motic, Wetzlar, Germany). The structure of the interdigitated capacitor of LC sensor and the microstructure of the BBT film (surface and cross section) were examined using the scanning electron microscopy SEM (JEOL JSM-6460LV, Jeol Ltd., Tokio, Japan).

## 3. Experimental Setup

**Figure 5:**
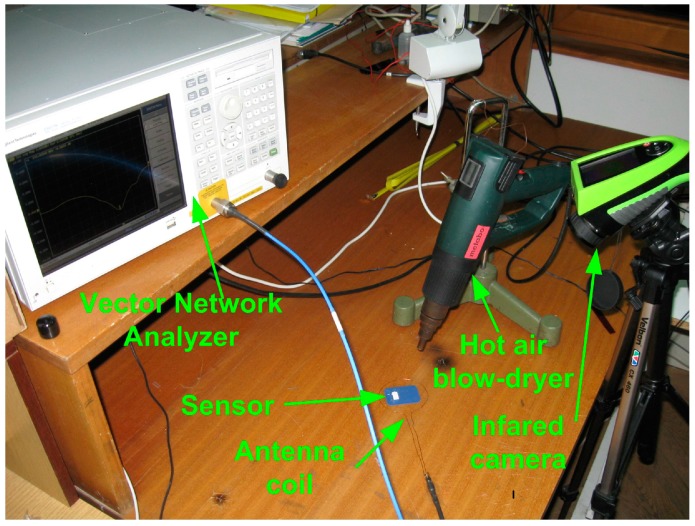
Experimental setup for measuring temperature (sensor, antenna coil, Vector Network Analyzer E5071B, hot air blow-dryer and Infrared Camera).

**Figure 6:**
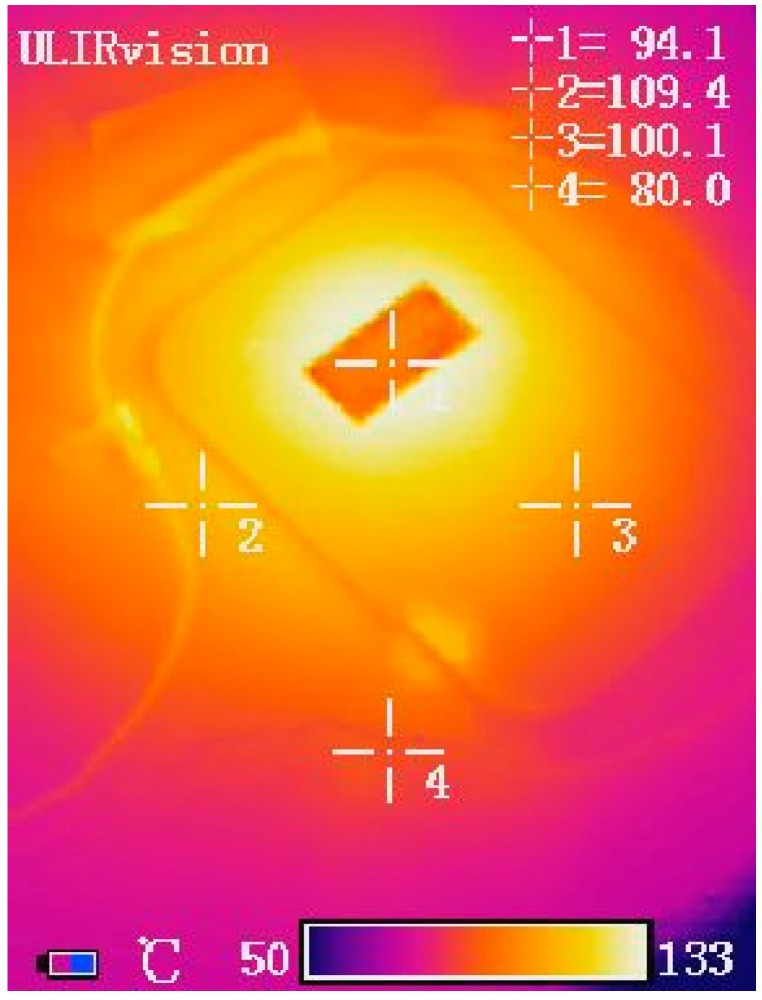
Image captured during the heating of the sensor using Infrared Camera.

*S*-parameters (scattering parameters) measurements were performed using an instrument Vector Network Analyzer E5071B, in the frequency range from 300 kHz to 8.5 GHz. In order to mesure *S*-parameters at elevated temperature, the sensor was heated using a hot air blow-dryer. In order to determine the exact temperature at which measurement was performed, Infrared Camera UlirVision TI1600 (Zhejiang ULIRVision Techology CO. Ltd., Hangzhou, China) was used. An infrared camera was chosen over a thermocouple for measuring temperature because it fitted better the experimental setup (Figure 5). Since the change in resonant frequency was correlated to the change in temperature of the BBT layer, it was important that that one was measured instead of the temperature of the surrounding atmosphere. Infrared camera was more suitable tool for such a task. Infrared camera that was used in experiment has accuracy of ±2 °C or ±2% in the temperature range −20–600 °C and it was calibrated before use. Figure 6 shows the appearance of the sensor during heating and the temperatures recorded using the Infrared Camera. For the purpose of the measurements, temperature was taken at the surface of the BBT layer of the sensor, indicated as point 3 in Figure 6. Heating of the sensor was carried out in the temperature range from 25 °C to 165 °C. The changes of |S11| parameter during the heating of the sensor were measured using both VNA and antenna coil placed around the sensor.

## 4. Results and Discussion

**Figure 7:**
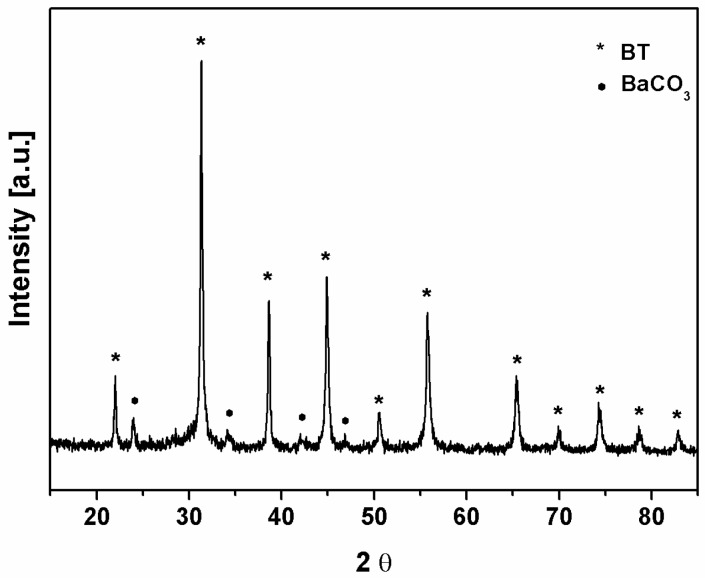
X-ray diffractogram of the BBT powder.

X-ray diffraction of the powder confirmed the presence of BaTiO_3_ tetragonal phase, since all the characteristic diffractions were observed (Figure 7). Additionally, the presence of small amount of BaCO_3_ was detected.

**Figure 8:**
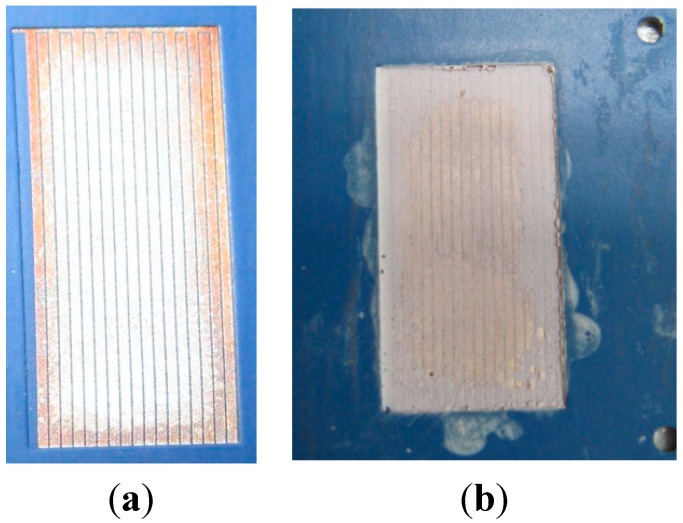
(**a**) The enlarged image of interdigitated capacitor before the deposition of the BBT film; (**b**) The visual appearance of the sensor after the deposition of the BBT film.

**Figure 9:**
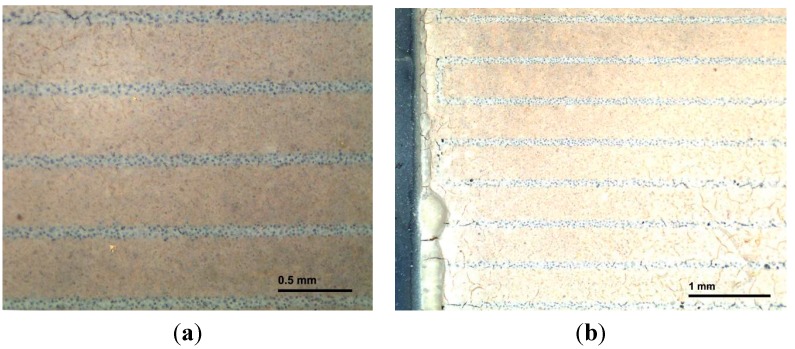
(**a**) Magnified pictures of the film thermally treated at 550 °C for 1 h; (**b**) Delamination at the edges of the interdigitated capacitor.

**Figure 10:**
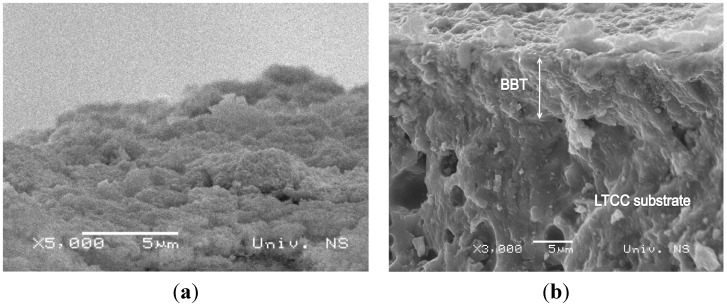
SEM micrographs of the BBT film: (**a**) 550 °C; (**b**) 800 °C, cross section.

The enlarged image of interdigitated capacitor before the deposition of the BBT film is shown in Figure 8. In order to check the appearance of the interdigitated capacitor after the deposition of the paste, *i.e.*, the structure and the uniformity of the BBT film, it was examined under the optical microscope. The surface of the film (Figure 9a) after the thermal treatment at 550 °C for 1 h in air appeared quite uniform, even though some cracks and delaminations at the edges of the interdigitated capacitor appeared. In order to avoid this, additional homogenization of the paste in the rotary mill and the optimization of the paste rheology should be considered. Delamination occurred at the edges of the interdigitated capacitor (Figure 9b), but this effect was considered not to have a negative impact on the sensor performances. The film thermally treated at 800 °C showed no peeling off the substrate, thus this temperature was selected as an optimal. In order to further investigate the microstructure and the adhesion of the film to the substrate, the BBT films were examined by SEM. SEM micrographs of the BBT films at LTCC ceramic substrate for the sensors thermally treated at 550 °C (Figure 10a) and 800 °C (Figure 10b) suggest quite uniform microstructure of the film, with the thickness of about 10 μm. Furthermore, from the cross section of the film thermally treated at 800 °C, it can be seen that the adhesion of the film to the substrate is very good, and that no delaminations or microstructure defects (e.g., pores, cracks) which would hinder the operation of the sensor, are present at the interface.

**Figure 11:**
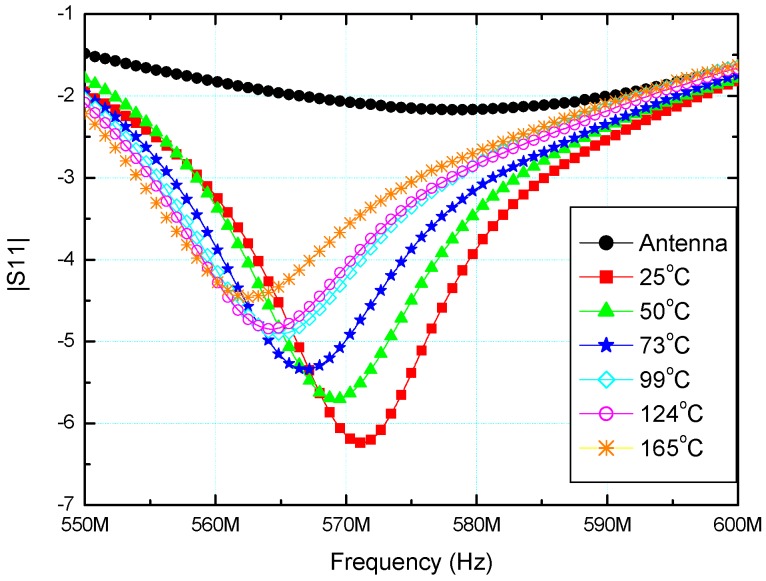
The parameter |S11| as a function of frequency and temperature.

**Figure 12:**
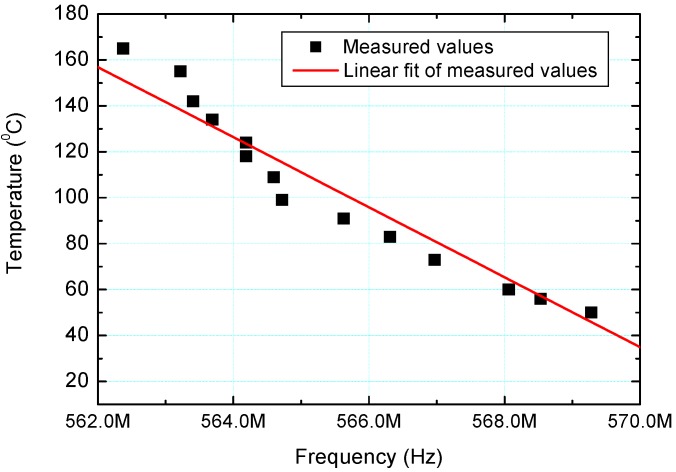
The temperature variation of the LC sensor as a function of resonant frequency.

Temperature dependence of the |S11| parameter of the sensor is shown in Figure 11. *S*-parameters are defined for a given frequency and system impedance, and vary as a function of frequency for any non-ideal network. Thus |S11| refers to the ratio of signal that reflects from port one for a signal incident on port one. By increasing the temperature ranging from 25 °C to 165 °C, |S11| parameter was simultaneously decreased from 571.06 MHz to 562.37 MHz. Total temperature change was 140 °C, while the change of the parameter |S11| was 8.69 MHz. Based on this, the sensitivity of the sensor has been calculated and it was equal to 62.07 kHz/1 °C, which was very high and indicated that the temperature measurements with presented sensor can be performed very accurately. As the temperature increased the peak of |S11| parameter decreased and shifted towards lower frequencies. This results from both the increased resistance of the inductive coil and increased conductivity of the insulating ceramic. Dependence of the resonant frequency of the sensor and variation of temperature is plotted in Figure 12. As can be seen, linearly fitted red line deviates slightly from the measured values (dark square symbols in Figure 12). Resonant frequency decreases with increasing of the sensor’s temperature. This results from the fact that relative permittivity for analyzed BBT increases with an increase of temperature up to some dielectric maximum. As a result the capacitance of the LC sensor increases and in accordance with the equation $f_{r}=1/\left(2\times \pi \times \sqrt{L\times C}\right)$, resonant frequency decreases. This is a consequence of the sensor’s capacitance increase. As the sensor’s temperature is increased, capacitance value of the fabricated passive LC sensor increases due to the rise of the permittivity of the BBT, since other capacitor parameters can be considered invariable. It is know that relaxor ferroelectrics, to which group belongs BBT, have broad and dispersive dielectric maximum. Accordingly, strong temperature dependence of permittivity was found in the particular temperature range for particular frequencies, as show in [13]. It is worth mentioning that resonant frequency belongs to the very high frequency range, thus measuring with some Vector Network Analyzer E5071B is not possible, because of that we used VNA for experimental characterization (which can cover frequency range up to 8.5 GHz). After the passive LC temperature sensor would cool down, the resonant frequency would return to the initial value, thus indicating that the permittivity value would return to its initial value. This means that the whole process is reversible and can be repeated many times (degradation and stress in material is a limitation).

## 5. Conclusions

In this paper, we have presented a wireless sensor for measuring temperature, based on the temperature dependence of permittivity of BBT. The presented sensor is quite simple for manufacturing. The advantage of the sensor presented in this paper is that the temperature measurement can be performed wirelessly and without contact. In addition, the sensor proposed has no problem with the lifetime of the battery since it is passive and does not require additional power. The sensor platform was manufactured using LTCC technology, and therefore it can be used at very high temperatures and in harsh environment. On the other hand, the BBT coated layer used is environmentally friendly, and presents a lead-free material with almost linear increase of permittivity in the presented temperature range. Based on our measurements one can conclude that the proposed LC passive wireless sensors could be used to measure temperature in a wide temperature range from 25 °C to 165 °C.
